# The experience of adolescence process among French teenager pregnancies: a mixed-methods study

**DOI:** 10.1080/17482631.2024.2386715

**Published:** 2024-08-04

**Authors:** Mireille Cosquer, Catherine Jousselme, Bruno Falissard, Aline Lefebvre

**Affiliations:** a CESP, U1018, Paris-Saclay University, Bicêtre University Hospital, Le Kremlin Bicêtre, France; b Academic Department, Foundation Vallée Hospital, Gentilly, France; c Human Genetics and Cognitive Functions, Institut Pasteur, UMR 3571 CNRS, University Paris Diderot, Paris, France

**Keywords:** Mental health, pregnancy, social support, mixed methods, adolescence

## Abstract

**Purpose:**

Teenage pregnancy remains a significant global public health concern worldwide. However, it presents a complex phenomenon in developed countries, carrying potential short- and long-term consequences for both mothers and children.

**Methods:**

This mixed method study used data from the French cross-sectional study “Portraits of adolescents”, which included 6000 girls aged between 13 and 17 years. The quantitative approach involved comparisons between a subgroup with an history of pregnancy and their peers, examining their lived-experience and mental health. The qualitative approach investigated the question “What does being a teenager mean for you?” specifically for the girls who reported an history of pregnancy.

**Results:**

Teenage pregnancies presented elevated rates of mental health disorders, including dark thoughts, depression, self-harm, participating in dangerous games, attempting suicide and increased use of psychoactive substances. With limited support, in comparison to their peers. The qualitative approach revealed three major themes: “being in action”, “a way of feeling”, and “quality of relationship”.

**Conclusion:**

This vulnerable subgroup of adolescents suggests the need for a coordinated multidisciplinary healthcare approach, given their limited parental and friend support, with a high risk of experiencing poor mental health. Additionally, these findings portray a “silent sufferer” population characterized by difficulties recognizing or managing emotions due to difficulties in expressing their emotional distress.

## Introduction

With 20% human beings today being teenagers, adolescence is a topic more addressed literature, as seen by the number of publications referenced in *Medline* which has more than doubled in the last twenty years until 2020, before abruptly decreasing last two years. In terms of development, the process of adolescence, which commences at puberty, is defined as the physical, hormonal and neurodevelopmental transformations before reaching adult maturity (Sawyer et al., 2012). Adolescents are considered as a vulnerable group, in reason of their specific brain immaturities (Sturman & Moghaddam, 2011). During adolescence, mental health conditions account for a significant proportion of disease burden (WHO, 2014). Depression is the leading cause of illness and disability among adolescents, and suicide is one of the top three causes of death. Various mental illnesses, including depression, schizophrenia, eating disorders and addictions, begin during adolescence. Furthermore, half of all mental health disorders in adulthood first appear before the age of 14, many of which go undetected and untreated (Sawyer et al., 2012).

Certain aggravating factors can increase adolescents’ vulnerability, leading to dangerous behaviours, that sometimes results in self-sabotage and/or psychiatric disorders (WHO Adolescent report, 2014). The relationships between personal, family, and social risk factors that promote the onset of behavioural disorders and the protective factors that help prevent them are still poorly understood.

In the 1990s, the United States established the Youth Risk Behavior Surveillance System as a routine data collection tool for tracking mental health and lifestyle indicators among adolescents. In France, in 1993, the National Institute of Health and Medical Research created the first school survey based on multiple perspectives of adolescents (Choquet & Ledoux, 1994). Following this initial epidemiological survey, a series of survey instruments have been regularly conducted in France under the impetus of the World Health Organization (WHO): the Health Behavior in School-aged Children survey (Currie et al., 2001), the ESPAD (European School Survey Project on Alcohol and Other Drugs) (Hibell et al., 2012), and the ESCAPAD (Health and Consumption Survey during the National Defence and Citizenship Day). While these diverse studies provide valuable indicators for prevention policies, they tend to concentrate on specific themes without fully addressing adolescents within the intricate context of their developmental process. Each high-risk behaviour is often examined in isolation (such as violence, substance use, or school bullying), possibly due to the involvement of various stakeholders. However, there are connections between high-risk behaviours, lifestyles, and adolescent mental health, underscoring the need for comprehensive studies on high-risk behaviours among adolescents in Europe (Hale & Viner, 2012). National-level data remains crucial for identifying local priorities and securing political commitment (Boerma & Abou-Zahr, 2005).

Quantitative epidemiological studies are widely used to study at risk adolescent populations, but their designs do not typically incorporate prevention strategies. The use of mixed methods, combining quantitative and qualitative approaches, is still limited in public health research in Europe (Creswell & Plano Clark, 2011; Guével & Pommier, 2012; Tashakkori & Teddlie, 2010). Nevertheless, these methods have gained popularity in recent years, particularly within healthcare systems, allowing researchers to analyse problems from multiple perspectives. According to some authors, this approach is especially suitable for understanding vulnerable populations (Gomez, 2014). The potential of this approach enables researchers to efficiently understand and explain complex issues prevalent in the adolescent population (Creswell & Plano Clark, 2011).

All these findings led us to propose an extensive multicenter study in France to better understand the complex relationships inherent in the adolescence process within the general population. We focused on a specific high-risk subgroup: teenage pregnancy, experiencing a tumultuous period characterized by physical, hormonal, and psychological changes, accompanied by a change in social status. The association between teenage pregnancy and mental health beyond the postpartum period remains unclear (Xavier et al., 2018). Engaging in risk behaviours is a normal aspect of adolescent development, including the exploration of sexuality. Nevertheless, this subgroup accumulates risk behaviours related to normal aspect of adolescent development, and early exploration of sexuality. For instance, depression is commonly observed among girls during adolescence and the postpartum period. Previous studies have identified perceived social support as a significant protective factor against teenage pregnancies (Dinwiddie et al., 2018; Peter et al.,^2017^).

In France, with the median age of first sexual intercourse in France around 17 years old (Bajos et al., 2016), fewer than 2% of mothers give birth before their twentieth birthday (Insee) since 2010, and the rate of induced abortions remains at 5 per thousand for adolescents under 18 years old in 2021 (https://drees.solidarites-sante.gouv.fr). Despite the 2001 law mandating sexuality education from elementary to high school, teenage pregnancy remains a complex phenomenon with potential short- and long-term consequences for both mothers and children. While studies identify factors associated with teenage pregnancies, very few explore adolescents’ own perceptions and behaviours in developed countries.

### Objective

The aim of this study was to describe the lived-experiences of the adolescents within a high-risk subgroup experiencing teenage pregnancies. It examined various aspects of their lives using a quantitative approach to explore their profiles (socio demographic, social support, physical health, sexuality, mental health), as alongside their self-perceptions and representations relative to adolescence through qualitative methods. Ultimately, the study seeks to propose effective prevention strategies in healthcare.

## Material and methods

### Study design

The “Portraits d’Adolescents” study was a cross-sectional, multicentre study conducted in autumn 2013 across schools in three diverse regions of France: Val de Marne (an urban department in the Paris area), Hautes Alpes (a semi-urban department in the Provence Alpes Côte d’Azur area), and the Poitou-Charentes region (a rural area). The study design and implementation were led by the Institut National de la Santé et de la Recherche Médicale (INSERM) in collaboration with other experts. A total of 134 schools, including middle schools, general high schools, vocational high schools, mixed-status high schools, agricultural high schools and rural training organizations, participated, encompassing 730 classes from grade 8 through to grade 12.

Questionnaires were self-administrated in classroom under the supervision of school staff. This study received approval from the National Advisory Ethics Committee and was conducted confidentially in compliance with CNIL (Commission Nationale de l’Informatique et des Libertés) guidelines (Protocol No. 912523).

For this study, the concurrent nested protocol, proposed by Creswell and Plano Clark (2011), was selected among various research methodologies. This approach integrates qualitative and quantitative methods concurrently, allowing them to inform each other throughout the research process. While the quantitative approach serves as the primary framework, this study aims to illustrate interconnections using a mixed-methods approach across a large adolescent sample. Qualitative analysis supplements traditional quantitative methods by exploring adolescents’ spontaneous discourse, addressing lesser—known aspects.

### Participants

A total of 15,235 participants aged 11 to 23 years were included, achieving a participating rate of 91.4%. The definition of adolescence, much like teenage pregnancy, remains unclear in the literature, with the WHO considering it as under 18 years, while in France, it is often considered under 20 years. In the article, we defined adolescence as under the age of majority and focused on a subsample of 6800 girls aged 13 to 17 years. We categorized participants into two groups: those with a history of pregnancy or abortion, identified by affirmative responses to questions “have you ever been pregnant?” or “have you ever had an abortion?” and those who responded negatively to both questions, categorized as non-pregnant peers.

### Measures

The anonymous questionnaire covered ten primary domains with a total of 348 questions, including 13 open-ended questions. Questions were selected based on their documented associations with the history of teenage pregnancy (Table I).

**Table I.** Quantitative approach questionnaire items.

Domain	Question
School/Social and parental support	What school year are you in?
	Have you ever repeated a year? *Never/Once/Twice or more*
	Currently, what do you think about school? *I like it a lot/I like it a bit/I don’t like it a lot/I dont like it at all*
	You would say that a school education is stressful *Yes/No*
	Generally, how satisfied are you with your relationship with your actual (real life) friends? *very satisfied/satisfied/neither satisfied nor dissatisfied/not very satisfied/not satisfied at all*
	How many friends do you have (Actual real life friends)? *None/2–4/5–10/>10*
	Generally, how satisfied are you with your relationship with your mother? very satisfied/satisfied/neither satisfied nor dissatisfied/not very satisfied/not satisfied at all
	Generally, how satisfied are you with your relationship with your father? *very satisfied/satisfied/neither satisfied nor dissatisfied/not very satisfied/not satisfied at all*
Physical health /self perception	How tall are you? What is your weight?
	Compared to other people of your age, would you say that your health is : *Not at all satisfactory/Unsatisfactory/Fairly satisfactory/Very satisfactory*
	Would you say your look is: *Feminine/Natural/Adolescent/Sexy/Sophisticated/Too masculine/Childish/Athletic/Neglected/Without specificity*
Sexuality (first sexual intercourse)	Have you ever had sex? *Yes/No*
	How old were you
	The first time you had sex was with: *a boy/a girl*
	How long had you been together? *a few years/a few months/a few days/just meet him*
	During this first time you had sex, did you (or your partner) use: *The pill/Condoms/Another contraception method/No contraception method*
	Were you in love with this partner? *No/Yes/I don’t know*
Mental health	Do you think that adolescence is an easy time? *No/Yes/Not always*
	Are you confident about the future? *No/Yes/Not always*
	Have you ever hurt yourself on purpose? *Never/Rarely/Quite often/Very often*
	Have you ever taken part in dangerous games? *Never/Rarely/Quite often/Very often*
	Have you ever thought that life was not worth living? *Yes/No*
	ADRS scale
	Are you currently seeing a psychiatrist or psychologist? Yes/No
Psychoactive substance use	Have you smoked cigarettes in the last 30 days? *No/Less than one cigarette a week/Less than one cigarette a day /1–5 cigarettes a day /6–10 cigarettes a day/11–20 cigarettes a day More than 20 cigarettes a day*
	How many times have you had cannabis? *(pot, a joint, hash, marijuana). In the last 30 days: 0 times 1–2 3–5 6–9 10–19 20–39 40+*
	How many times have you had an alcoholic drink? *In the last 30 days: 0 times 1–2 3–5 6–9 10–19 20–39 40+*
	Think back over the last 30 days. How many times did you have five “drinks” or more on one occasion? *None 1 time 2 times 3–5 times 6–9 times 10 times or more*

- Separation and family conflicts. School education was assessed via school level or failure to proceed to the next grade, school well-being. Parental and friend’s support was considered for responses “very satisfied” and “satisfied”; in contrast “neither satisfied nor dissatisfied, “not very satisfied”, or “not satisfied at all” were classified as without support and by the number of friends reported.
- Circumstances of first sexual intercourse were: early sexual intercourse (first sexual intercourse under 13 years), partners’ gender, length and love relationship, contraception use.
- Variables related to physical health included Body Mass Index (calculated from reported weight in kilograms and height in centimetres) and a dichotomous measure of self- rated health (fair or poor) (Breidablik, 2009). Mental health was assessed by the presence of dark thoughts, a history of previous suicide attempts, participation in dangerous games, experiencing self-harm, considering adolescence as a difficult period, confidence about the future, and receipt of psychological support. Additionally, the severity of depression was measured using the French version of the Adolescent Depression Rating Scale (Revah-levy, 2007), which was divided into three levels: No depression (score below 3), sub depression (score between 3 and 5), and severe depression (score of 6 or more). Specific risk indicators related to psychoactive substance were evaluated as follow: intensive smoking (at least ten cigarettes per day), regular alcohol consumption (at least 10 times during the last 30 days), regular binge drinking (at least 5 glasses on one occasion in the last 30 days), and regular cannabis use (at least 10 times in the last 30 days).

Additionally, an open-ended question was used for the qualitative analysis on the specific theme under investigation: “What does being a teenager mean to you?”.

### Data analyses

First, a quantitative approach was employed, where descriptive analysis was conducted to explore teenage pregnancies and adolescent girls, using bivariate analysis (chi-square). **We hypothesis that teenage pregnancy subgroup is more vulnerable than their peers, because of cumulative risks, and** used the Bonferroni correction due to the multiple tests, and the significance level was set at 0.001. Analyses were performed using SAS version 9.4.

Second, a thematic analysis (Braun et al., 2014) was used to examine the open-ended question data for the teenage pregnancies’ subgroup. This method allows the identification, analysis and reporting of themes within the data. Our thematic analysis was data-driven and employed an inductive approach, meaning the data was coded without reference to theoretical notions or researcher preconceptions.

We summarized the different stages of our thematic analysis:

1. code the verbatim by making notes corresponding to the fundamental units of meanings make conceptual
2. notes through processes of condensation, abstraction, and comparison of the initial notes
3. identify initial themes
4. identify recurrent themes across transcripts.

Regarding data handling, we extracted each response for the open-ended question “What does being a teenager mean for you? for the selected population of the pregnancy group. We uploaded this collection into software for qualitative analyses (N’Vivo version 12).

Two researchers team members (MC, CJ) coded line by line independently, and then exchanged coding outputs for cross-checking the coding quality. In the case of disagreements or appeals to re-code, members initiated coding meetings and discussed problematic codes until consent in the final output of coding was achieved. Moreover, the results were regularly discussed during research group meetings, with the presence of a third researcher (IC). In cases of disagreements, discussions continued until a consensus was reached. Thematically similar codes were associated into groups and sub-groups regarding responses of students The final output of the coding process and thematic code grouping was approved by the researchers. Afterwards, we extracted core themes, subthemes, contexts and quotations regarding perceived perceptions of students.

**Figure 1.**
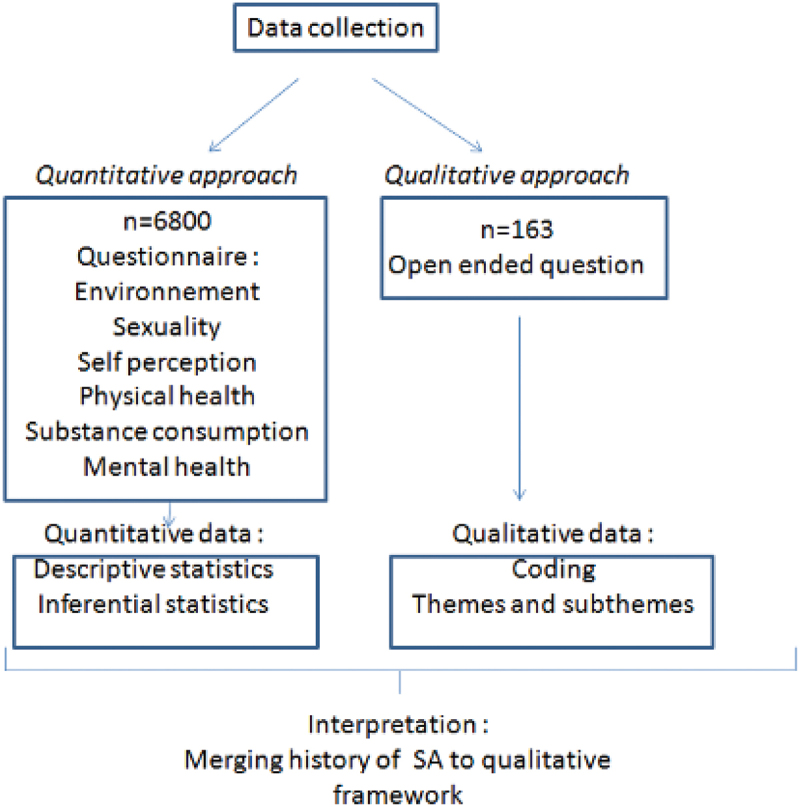
Steps of the analysis.

Extracts of the transcripts were selected to illustrate the described themes. Verbatim accounts were translated into English solely for the purposes of this article. Thirdly, we analysed these verbatims with consideration of whether they had been involved in suicidal acts. Figure 1 outlines the various steps of the analysis.

## RESULTS

### Population

Out of 6,800 girls aged 13 to 17, 163 (2.4%) were identified as having experienced teenage pregnancy (133 reported pregnancies, while 30 reporting abortions without prior pregnancy). Within the pregnant subgroup, 118 girls opted for abortions, resulting in a net total of 43 pregnancies (and 2 no responses). Characteristics are presented in Table II.

**Table II.** Socio demographic characteristics.

	History of pregnancy	Peers	*p*
	n = 163	n = 6637	
**Age (*n* = 6800)**	nb (%)	nb (%)	
mean ± sd	15.8 ± 1.2	15.2 ± 1.4	<0.0001
13	10 (6.1)	1080 (16.3)	
14	15 (9.2)	1145 (17.2)	
15	30 (18.4)	1442 (21.7)	
16	49 (30.1)	1561 (23.5)	
17	59 (36.2)	1409 (21.2)	
**Level of school (*n* = 6800)**			
Middle	40 (24.5)	2484 (37.4)	<0.001
High	123 (75.5)	4153 (62.6)	
**Grade repetition (*n* = 6792)**			
No	84 (51.5)	5078 (76.6)	<0.0001
Yes	79 (48.5)	1551 (23.4)	
**Region (*n* = 6800)**			<0.0001
Urban	36 (22.1)	1988 (29.9)	
Semi-urban	61 (37.4)	3173 (47.8)	
Rural area	66 (40.5)	1476 (22.2)	
**Professional activity of father (*n* = 6580)**			
Yes	112 (72.3)	5399 (84)	<0.0001
No	43 (27.7)	1026 (16)	
**Professional activity of mother (*n* = 6677)**			
Yes	122 (75.8)	5269 (80.9)	0.11
No	39 (24.2)	1247 (19.1)	
**Parents’education level (*n* = 6800)**			
None with high education level	99 (60.7)	3320 (50)	0.02
At least one with high education level	42 (25.8)	2378 (35.8)	
Don’t know for both parents	22 (13.5)	939 (14.1)	
**Family conflict (*n* = 6756)**			
Never	7 (4.3)	521 (7.9)	<.0001
Rarely	54 (33.3)	3127 (47.4)	
Quite often	47 (29)	2000 (30.3)	
Very often	54 (33.3)	946 (14.3)	
**Live with both parents (*n* = 6687)**			<0.0001
Yes	59 (37.3)	3826 (58.6)	
No	99 (62.7)	2703 (41.4)	

Teenage pregnancies were older (mean age of 15.8), more frequent at high school (75.5%) and 48.5% reported failure to proceed to the next grade. Adolescents in this group were more likely to live in rural areas (40.5%), and the majority (62.7%) did not live with both parents. Additionally, there were more family conflicts reported, and a higher proportion of parents had lower educational educational level.

### Quantitative results

As shown in Table III, among the teenage pregnancies subgroup, obesity was more prevalent (8% vs 2.3%, p < 0.0001), as a lower self-rated health (28.8% vs 11.3%, p < 0.0001). They also reported riskier sexual behaviour for their first sexual experience: 30.1% experienced this before the age of 13 years (vs 13.2%, p < 0.0001), 26% declared not using any contraception method (vs 10.5%, p < 0.0001), and 12.8% reported meeting their partner just prior to intercourse (vs 5.8%, p < 0.001).

**Table III.** Physical health and sexuality.

	History of pregnancy n = 163	Peers n = 6637	*p*
	nb (%)	nb (%)	
*Physical health*			
**BMI (*n* = 6799)**			
underweight	13 (8)	506 (7.6)	0.87
normal	127 (77.9)	5623 (84.7)	0.02
overweight	10 (6.1)	352 (5.3)	0.64
Obesity	13 (8)	155 (2.3)	<.0001
**Self-rated health (*n* = 6760)**			<0.0001
Low	47 (28.8)	749 (11.3)	
High	116 (71.2)	5848 (88.7)	
*Sexuality (first sexual relation)*			
**Age first sexual relation** ≤**13 years (*n* = 2059)**			<0.0001
Yes	47 (30.1)	251 (13.2)	
No	109 (69.9)	1652 (86.8)	
**Type of sexual experience (*n* = 1969)**			
heterosexual	154 (97.5)	1761 (97.2)	0.87
homosexual	4 (2.5)	50 (2.8)	
**Relationship since (*n* = 2015)**			
a few years	26 (16.7)	235 (12.6)	0.15
a few months	93 (59.6)	1245 (67)	0.06
a few days	17 (10.9)	271 (14.6)	0.21
just meet him	20 (12.8)	108 (5.8)	<0.001
**Contraception method (*n* = 1980)**			
the pill	30 (19)	415 (22.8)	0.27
no contraception method	41 (26)	191 (10.5)	<0.0001
condoms	113 (71.5)	1588 (87.2)	<0.0001
another contraception method	4 (2.5)	22 (1.2)	0.16
**In love with this partner (*n* = 2063)**			0.24
No	24 (15.1)	205 (10.8)	
Yes	121 (76.1)	1511 (79.4)	
Don’t know	14 (8.8)	188 (9.9)	

Parental and social support findings are summarized in Table IV. Teenage pregnancies reported lower parental support compared to their peers, with less support from their mothers (66.9% vs 79.6%, p < 0.0001), and fathers (46.1% vs 64.5%, p < 0.0001). Although friend’s support did not reach statistical significance, teenage pregnancies reported more internet’s friends (37.6% vs 24% p < 0.001) and significantly lower satisfaction with school (16.6% vs 6%, p < 0.0001). This subgroup was also more likely to consider their look feminine and sexy (respectively 82.2% and 34.4% vs 67.7% and 10.2%).

**Table IV.** Support social and self perception.

	History of pregnancy n = 163	Peers n = 6637	*p*
	nb (%)	nb (%)	%
**Mother’s support (*n* = 6681)**			<0.0001
No	53 (33.1)	1333 (20.4)	
Yes	107 (66.9)	5188 (79.6)	
**Father’s support (*n* = 6517)**			<0.0001
No	83 (53.9)	2260 (35.5)	
Yes	71 (46.1)	4103 (64.5)	
**Friend’s support (real life) *n* = 6705**			
No	22 (13.8)	540 (8.2)	0.01
Yes	137 (86.2)	6006 (91.8)	
**Number of friends real life (*n* = 6754)**			0.0005
0–4	35 (21.5)	810 (12.3)	
>5	128 (78.5)	5781 (87.7)	
**Number of internet’s friends (*n* = 6506)**			<0.0001
0–4	96 (62.4)	4827 (76)	
>5	58 (37.6)	1525 (24)	
*School*			
**Like school (*n* = 6778)**			
A lot	21 (12.9)	988 (14.9)	0.46
a little	81 (49.7)	3654 (55.2)	0.16
not so much	34 (20.9)	1577 (23.8)	0.37
Not at all	27 (16.6)	396 (6)	<0.0001
**School is stressful (*n* = 6485)**			0.18
Yes	117 (75)	4430 (70)	
No	39 (25)	1899 (30)	
*Self perception*			
**Your look is**			
Feminine	134 (82.2)	4494 (67.7)	<0.0001
Natural	76 (46.6)	3637 (54.8)	0.04
Adolescent	73 (44.8)	3589 (54.1)	0.02
Sexy	56 (34.4)	680 (10.2)	<0.0001
Sophisticated	25 (15.3)	629 (9.5)	0.01
Too masculine	6 (3.7)	342 (5.2)	0.40
Childish	2 (1.2)	172 (2.6)	0.28
athletic	5 (3.1)	330 (5)	0.27
Neglected	6 (3.7)	256 (3.9)	0.91
Without specificity	33 (20.2)	1499 (22.6)	0.48

Mental health and substance use among girls are presented in Table V. Teenage pregnancies subgroup more reported significantly higher rates of dark thoughts (67.9% vs 47.4%, *p* < 0.0001), depression (39.4% vs 16.2%, *p* < 0.0001), at least one suicide attempt (50.9% vs 14.5%, *p* < 0.0001), self-harm (35.8% vs 11.7%, *p* < 0.0001), and participation in dangerous games (24.1% vs 4.8%, *p* < 0.0001) compared to their peers. Only 20.2% in this subgroup received psychological follow up. Additionally, 19,1% drank regularly vs 3.8%, 22.9% consumed regularly cannabis vs 4.5% and smoked intensively 60.1% vs 18.4%.

**Table V.** Mental health and psychoactive substance use.

	History of pregnancy n = 163	Peers n = 6637	
*Mental health*	nb (%)	nb (%)	p
**Considering adolescence as a difficult period (*n* = 6756)**			0.01
No	64 (39.3)	2075 (31.5)	
Yes	21 (12.9)	610 (9.2)	
Not always	78 (47.8)	3908 (59.3)	
**Confidence in future (*n* = 6745)**			0.04
No	34 (20.9)	927 (14.1)	
Yes	62 (38)	2578 (39.2)	
Not always	67 (41.1)	3077 (46.7)	
**Experience of self-harm (*n* = 6772)**			<0.0001
No	104 (64.2)	5836 (88.3)	
Yes	58 (35.8)	774 (11.7)	
**Participating in dangerous games (*n* = 6765)**			<0.0001
No	123 (75.9)	6284 (95.2)	
Yes	39 (24.1)	319 (4.8)	
**Dark thoughts (*n* = 6725)**			<0.0001
No	51 (32.1)	3453 (52.6)	
Yes	108 (67.9)	3113 (47.4)	
**Depression (*n* = 6560)**			<0.0001
No	97 (60.6)	5363 (83.8)	
Yes	63 (39.4)	1037 (16.2)	
**Suicide attempt (*n* = 6744)**			<0.0001
No	80 (49.1)	5628 (85.5)	
One	40 (24.5)	664 (10.1)	
Several	43 (26.4)	289 (4.4)	
**Psychological follow up (6725)**			<0.0001
No	130 (79.8)	6016 (91.7)	
Yes	33 (20.2)	546 (8.3)	
*Substance consumptions*			
**Daily tobacco use (*n* = 6783)**			<0.0001
No	65 (39.9)	5401 (81.6)	
Yes	98 (60.1)	1219 (18.4)	
**Regularly marijuana use (*n* = 6554)**			<0.0001
No	121 (77.1)	6112 (95.5)	
Yes	36 (22.9)	285 (4.5)	
**Regularly alcohol use (*n* = 6498)**			<0.0001
No	127 (80.9)	6098 (96.2)	
Yes	30 (19.1)	243 (3.8)	
**Regularly binge drinking use (*n* = 6730)**			<0.0001
No	146 (89.6)	6496 (98.9)	
Yes	17 (10.4)	71 (1.1)	

### Qualitative results

A total of 86.5% girls in the pregnant group answer to the question “What does being a teenager mean for you?”. Three main themes emerged from their responses: to be in action (*n* = 70), a state of being (*n* = 34), relationships with others (*n* = 23). Themes, subthemes and examples are presented in Table VI.

**Table VI.** Themes, subthemes and examples.

Themes	Subtheme	Exemple
**Theme 1**	Subtheme n°1.1: Enjoy the present moment (*n* = 49)	*“For me, being a teenager means having fun, living…” (16 years old)*
	Subtheme n°1.2: Preparing one’s future (*n* = 25)	*“To enjoy life, but at the same time, work because that’s where everything happens.” (16 years old)*
	Subtheme n°1.3: Engaging in risky behaviours (*n* = 11)	*“Having a level of maturity for level appropriate for one’s age*, *making only foolish mistakes*, *not knowing knowing many things.” (15 years old, Suicidal Attempt)*
		*“Learning to grow up, discover many things*, *recognize one’s limits*, *party without caring about anything*. *Drinking to exceed one’s limits, smoking until nothing makes sense anymore.” (17 years old, Suicidal Attempt)*
		“*Not mastering one’s hormones. Becoming mature. Discovering one’s sexuality. Exploring one’s own limits*, *indulging in excesses.” (17 years old, without Suicidal Attempt)*
		“*Smoking, drinking, sexual intercourse*, *partying*, *drugs, police, prison.” (14 years old, Suicidal Attempt)*
**Theme 2**	Subtheme n°2.1: Identity construction.	“*Adolescence is the time when one discovers oneself*, *when one sees who they really are. Adolescence is crucial for becoming an adult.” (17 years old)*
	Sub-theme n°2.2: Carefreeness.	*“Because I am like everyone else, and I see life through rose-colored glasses.”* (17 years old without *Suicidal Attempt)*
	Sub-theme n°2.3: Difficult period.	*“A period in life where problems seem to pile up.” (17 years without Suicidal Attempt)*
		“*To feel uncomfortable in one’s own skin, to waste time learning things that don’t really matter. To see one’s family torn apart and realize that childhood was just an illusion, that life is not so beautiful, not so simple.” (17 years old, Suicidal Attempt)*
		*“It’s being between childhood and the real world.” (13 years old, Suicidal Attempt)*
		*“The period when we discover the hidden side of life that our parents didn’t show us when we were children.” (17 years old, Suicidal Attempt)*
		*“A person who realizes how terrible the world is and discovers that happiness is just a pretty lie served to children to give them false hopes.” (14 years old, Suicidal Attempt)*
		*“Left the world of children, witnessing realities of life, whether good or bad. Simply growing up, building oneself, having fun.” (17 years, without Suicidal Attempt)*
**Theme 3**	Sub-theme n°3.1: To be in a relationship of trust.	*“Being a teenager is having friends to play with*, *thinking like everyone else but without thinking too much.” (15 years old, without Suicidal Attempt)*
		*“A teenager is a mature person who dresses well, a kind person, but above all*, *respectful of others.” (16 years old, Suicidal Attempt)*
	Sub-theme n°3.2: To have conflicted relationships	*“Living with parents, doing mischief, and doing everything to make sure they never find out.” (16 years old, without Suicidal Attempt)*
		“*Tantrums for nothing*, *without any reason, just to be heard.” (16 years old)*
		“*Throwing tantrums, getting upset for no reason, still being a child, not taking responsibility for anything.” (17 years old)*
		*“Throwing tantrums at our parents for no reason, trying to be like others, and growing up too.” (15 years old)*

### Theme 1: to be in action

In this theme, girls enumerate numerous verbs, evoking impulsiveness. Girls describe their daily lives and express a wide range of activities. Firstly, the majority emphasize the enjoyment of life and having fun positively; secondly, they highlight the importance of the education system; finally, a few mentions engaging in risky behaviours.

- *Subtheme n°1.1: Enjoy the present moment (n = 49)*

Their responses are based on the enjoyment of the present moment, acting without constraints, engaging in various experiences (without the notion of capitalization).

For me, being a teenager means having fun, living… (16 years old)

- *Subtheme n°1.2: Preparing one’s future (n = 25)*

Future-oriented, girls mentioned investing in work and education, capitalizing on experience, preparing and learning.

To enjoy life, but at the same time, work because that’s where everything happens. (16 years old)

- *Subtheme n°1.3: Engaging in risky behaviours (n = 11)*

Overall, the perception of risk is not highly prominent, occurring only 11 times. There isn’t a predominant risk but rather concepts of multiple and cumulative risks.

- To do stupid things, to misbehave:

Having a level of maturity for level appropriate for one’s age, making only foolish mistakes, not knowing knowing many things. (15 years old, Suicidal Attempt)

- Discovering one’s limits, not having too many limits, recognizing one’s limits.

Learning to grow up, discover many things, recognize one’s limits, party without caring about anything. Drinking to exceed one’s limits, smoking until nothing makes sense anymore. (17 years old, Suicidal Attempt)

- Not mastering one’s sexuality.

Not mastering one’s hormones. Becoming mature. Discovering one’s sexuality. Exploring one’s own limits, indulging in excesses. (17 years old, without Suicidal Attempt)

- Consumption of psychoactive substances:

Smoking, drinking, sexual intercourse, partying, drugs, police, prison. (14 years old, Suicidal Attempt)

### Theme 2: a state of being

Girls perceived adolescence as a feeling based shaped by their experiences: as a stage of self-construction, a period of carefreeness, or a time of challenges.

- *Subtheme n°2.1: Identity construction.*

Exploration, self-discovery, self-building, questioning, and decision-making were reported by ten girls.

Adolescence is the time when one discovers oneself, when one sees who they really are. Adolescence is crucial for becoming an adult. (17 years old)

- *Sub-theme n°2.2: Carefreeness.*

The sensation of carefreeness or lightness was mentioned by a small number of girls (*n* = 7)

Because I am like everyone else, and I see life through rose-colored glasses. (17 years old without Suicidal Attempt)

- *Sub-theme n°2.3: Difficult period.*

This period was portrayed with negativity or challenges (*n* = 9).

A period in life where problems seem to pile up. (17 years without Suicidal Attempt)

To feel uncomfortable in one’s own skin, to waste time learning things that don’t really matter. To see one’s family torn apart and realize that childhood was just an illusion, that life is not so beautiful, not so simple. (17 years old, Suicidal Attempt)

Nine girls depicted this period as an awakening to reality or truth, marked by disenchantment and disillusionment.

It’s being between childhood and the real world. (13 years old, Suicidal Attempt)

The period when we discover the hidden side of life that our parents didn’t show us when we were children. (17 years old, Suicidal Attempt)

A person who realizes how terrible the world is and discovers that happiness is just a pretty lie served to children to give them false hopes. (14 years old, Suicidal Attempt)

Left the world of children, witnessing realities of life, whether good or bad. Simply growing up, building oneself, having fun. (17 years, without Suicidal Attempt)

### Theme 3: relationships with others

The third theme reflects relationships with others: whether they feel confident or not.

- *Sub-theme n°3.1: To be in a relationship of trust.*

Respect, friendship, fun were qualities mentioned by the girls.

Being a teenager is having friends to play with, thinking like everyone else but without thinking too much. (15 years old, without Suicidal Attempt)

A teenager is a mature person who dresses well, a kind person, but above all, respectful of others. (16 years old, Suicidal Attempt)

- *Sub-theme n°3.2: To have conflicted relationships*

The mentioned relationships are characterized by distrust and disappointment.

Living with parents, doing mischief, and doing everything to make sure they never find out”. (16 years old, without Suicidal Attempt)

Additionally, throwing tantrums were reported by three girls.

Tantrums for nothing, without any reason, just to be heard. (16 years old)

Throwing tantrums, getting upset for no reason, still being a child, not taking responsibility for anything. (17 years old)

Throwing tantrums at our parents for no reason, trying to be like others, and growing up too. (15 years old)

Some subthemes were associated with a lower frequency of suicidal attempt (SA): sub-theme 1.2 (Preparing for the future), subtheme 2.1 (Identity construction) with no reported occurrences of SA, and subtheme3.1 (Being in a relationship of trust). Conversely, other subthemes were more prevalent among girls with reported SA: subtheme 3.2 (Having conflicted relationships), and subtheme 2.3 (challenging period) except if the presence of other subthemes as subtheme 2.1 (identity construction) or subtheme 1.1 (enjoy the present moment was associated

## Discussion

The strengths of this mixed-method study allow us to delve into the intricate adolescent process within a specific subgroup with a history of pregnancy, from the general population rather than in institutional settings. This approach is unique, encompassing a substantial number of students from various types of high schools and diverse geographical areas in France. The quantitative aspect highlights that teenagers with a history of pregnancy constitute a particularly vulnerable group, with a significant proportion experiencing depression (39.4% compared to 16.2%). While adolescence is widely recognized as a vulnerable period associated with a high risk of developing mental health disorders, pregnancy introduces an additional stress factor, contributing to a higher incidence of post-partum depression (25% vs. 10% in adult mothers) (Dinwiddie et al., 2018). Beyond depression, our findings indicate a correlation between a history of pregnancy and increased incidences of mental health risks such as self-harm, suicide attempts, dark thoughts, and participation in dangerous games. This association is consistent with co-occurring substance use disorders. These results align with the correlations identified between substance-related disorders, particularly alcohol and tobacco use, and adolescent pregnancy, emphasizing the need for integrated approaches to prevent and reduce alcohol and tobacco use among pregnant girls (Bottorff et al., 2014). Diagnosing depression in adolescence can be complex, and notably, only 24% of pregnant girls who have attempted suicide express optimism about their future, compared to 53% of those who have not attempted suicide.

Furthermore, they frequently lack adequate psychological support (only 20%), have limited family and social support as protective factors, and often experience heightened family conflicts (33%) coupled with less parental support. Reid and Meadows-Oliver (2007) similarly reported this association, indicating that increased levels of family conflicts, diminished social support, and low self-esteem were linked to higher rates of depressive symptoms among adolescent mothers in the first year postpartum. During adolescence, social support is recognized for its role in enhancing mental health. In our results, adolescents with a history of pregnancy reported a double sense of loneliness, in addition to their family difficulties; they have fewer real-life friends and more internet friends than their peers. One possible explanation for this disparity could be suggested with a 48.5% repetition grade. On the other hand, they don’t seem to be opposed to school, as no association was found with stress at school. Spence’s (2008) reinforce these concerns, revealing an increased risk of academic underachievement and insufficient social support among teenage mothers. Furthermore, Peter’s et al. (2017) illustrated that perceived social support acts as a protective factor against anxiety disorders, exerting a positive influence on their overall mental well-being. However, drawing definitive conclusions regarding the long-term mental health risks associated with adolescent pregnancy remains a challenge due to the absence of prospective cohorts of adolescent mothers (Xavier et al., 2018).

The qualitative aspect of our study further enhanced these results, portraying these young girls as a “silent suffering” population with limited avenues to express their distress. They struggle with recognizing or managing emotions, showing signs of denial or internal division, as only 11% acknowledge their potential state of distress. This discrepancy from the quantitative results suggests low verbalization abilities, with only 20% seeking help from a psychologist or psychiatrist. Therefore, the challenge lies in remaining vigilant and providing assistance to this silent population at high risk for psychiatric issues, aiming to prevent recurrent suicide attempts, pregnancies, abortions, and other risky behaviours. It is also noteworthy that certain themes are limitedly mentioned: sexuality (only mentioned 14 times out of 163), body image (13 times out of 163), and pregnancy (not mentioned at all). Could pregnancy be perceived as a “socially ashamed” theme by adolescent? The relationship with time (ability to project oneself into the future, transitioning from childhood, enjoying the present moment) is a subjective concept that is challenging to capture, as it is only indirectly perceived. Moreover, lived-experiences of adolescence reported considerable heterogeneity, with a gradient in the perception of the intricate process. These perceptions range from more elaborated and reflective to less developed, varying across different age groups.

Analysis based on the presence of suicide attempts revealed a complex interplay of factors. Isolated positive subthemes such as being in a trusting relationship and having future projection, appeared to be protective. Additionally, when a positive subtheme like identity construction coexisted with negative subthemes, it still demonstrated some protective effects. Conversely, negative subthemes alone, such as conflictual relationships and the challenges of adolescence, were more predictive of suicide attempts. Furthermore, the relationship with time emerged as a significant factor.

A Canadian study using a mixed-methods approach assessed the health perceptions of adolescents, highlighting differences from adult perceptions and contributing to the development of effective health promotion strategies (Michaelson et al., 2016).

Our study has certain limitations that need to be taken into account. Firstly, as with any cross-sectional study, we were unable to establish causality. Secondly, the study population is not a representative sample, and caution should be exercised when attempting to generalize the results to the entire national context. Similar to other school-based studies, this research excludes children who do not attend school, who constituted approximately 16% of 15 to 19-year-olds in France in 2012, as reported by the Organisation for Economic Cooperation and Development. This exclusion may improve the accuracy of assessing the health status of children under the age of 16. Patton et al. (2012) suggests implementing innovative data collection strategies targeted at socially marginalized youths, including those not enrolled in school.

Additionally, the study was conducted in 2013, and behaviours may have changed, especially with the use of new social networks and different operating modes. Thirdly, this material lacks information regarding the context of pregnancy, including timing, partner support, the presence of the child with her mother, and other influential factors such as ethnicity or cultural aspects. It also does not offer a longitudinal perspective on adolescents, which could have allowed for a more in-depth exploration of the emergence and evolution of psychological disorders.

Lastly, data collection through self-administered questionnaires has inherent limitations for interpretation. The ADRS scale approximates a psychiatric diagnosis, and qualitative data, such as a few words collected, raises concerns regarding the understanding of open-ended questions. For instance, it is unclear whether the responses reflect their personal perceptions or general opinions. Although analysing such text in a self-administrated questionnaire is unconventional and ensuring comprehension of the questions is challenging, it provides a unique perspective on the adolescent themselves. While it would have been interesting for the qualitative analysis to encompass the entire population, logistical constraints made this impractical.

The primary strengths of this study lie in its substantial sample size and the comprehensiveness and reliability of the collected variables. Notably, studies of this magnitude involving text variables are relatively scarce due to the associated high analytical costs. Given the contrasting perceptions of adolescent lived-experiences between adults and youths, the qualitative approach, which allows access to the adolescents’ narrative identities in their own words, is particularly justified. This is especially significant considering that very few qualitative studies focus on adolescent girls. Consequently, the complementary nature of the qualitative approach proves valuable from multiple perspectives. Our findings align with the Cochrane review of Oringanje et al. (2016), emphasizing the effectiveness of interventions that integrate education and contraception promotion. This highlights the crucial role of engaging diverse professionals in supporting these vulnerable young girls.

## Conclusion

Girls with a history of pregnancy face a significantly elevated risk of mental health disorders than their peers, with a limited parental and social support. These findings underscore the importance and urgent necessity of adopting a comprehensive interdisciplinary approach in clinical practice with pregnant adolescent, considering not only their verbal expressions but also their mental state. Such an approach should involve a coordinated multidisciplinary healthcare approach, including addiction specialists, psychologists, and gynaecologists. Furthermore, our findings suggest the importance to explore more factors associated with suicide attempts in this “silent sufferer” population. Recent guidelines (de Santé, 2024) in France recommend systematically offering psychologist follow-up to pregnant adolescents.

## Supplementary Material

bio note.docx
