# Supplementary material for: The experience of adolescence process among French teenager pregnancies: a mixed-methods study
Source: Int J Qual Stud Health Well-being. 2024 Aug 4;19(1):2386715. doi: 10.1080/17482631.2024.2386715 (PMC11302457; doi:10.1080/17482631.2024.2386715)
Supplement: bio note.docx [file ZQHW_A_2386715_SM4306.docx]

- Mireille Cosquer has been working as a psychologist and statistician in the academic department of Fondation Vallée since 2013. She is also a member of the Moods Center (CESP- -Univ. Paris-Saclay, French National Institute of Health and Medical Research INSERM, Univ. Versailles Saint-Quentin-en-Yvelines UVSQ). Her work involves leading studies on the experiences of parents with sick children and on the adolescent process, particularly on the context of the multicenter epidemiological survey 'Portraits of Adolescents'.
- Dr Aline Lefebvre holds the position of Associate Professor - Hospital Practitioner in child and adolescent Psychiatry at the academic department of Fondation Vallée (Hospital of Child and Adolescent Psychiatry) and the Faculty of Medicine, University Paris-Saclay, Le Kremlin-Bicêtre since 2023. She specializes in neurodevelopmental disorders and leads various European research projects at UNIACT Neurospin – INSERM UMR 1129, CEA, Saclay.
- Professor Catherine Jousselme is a Professor of Child and Adolescent Psychiatry at Paris Sud University. She serves at the head of a significant child and adolescent psychiatry department in Gentilly, Fondation Vallée, and collaborates closely with pediatric services at Bicêtre University Hospital. Additionally, she is a member of the MOODS Center (CESP- Univ. Paris-Saclay, French National Institute of Health and Medical Research INSERM, Univ. Versailles Saint-Quentin-en-Yvelines UVSQ). Professor Jousselme leads studies on the experiences of parents with sick children, on the adolescent process (multicenter epidemiological survey 'Portraits of Adolescents'), and on self-esteem and creativity in adolescence. She is the author of numerous works.
- Professor Bruno Falissard has held the position of Professor of Public Health at Université Paris-Saclay since 2002. He is a child psychiatrist and serves as the Director of the [Centre for Epidemiology and Population Health (CESP - Univ. Paris-Saclay, French National Institute of Health and Medical Research INSERM, Univ. Versailles Saint-Quentin-en-Yvelines UVSQ)](https://cesp.inserm.fr/en). With a background in mathematics and psychiatry, and as a member of the French Academy of Medicine, Professor Falissard focuses his research on the epistemology and research methodology in medicine. Throughout his career, he has dedicated himself to advancing the understanding and treatment of mental health issues, particularly in the field of child and adolescent psychiatry. His extensive research has played a crucial role in shaping evidence-based practices and policies aimed at enhancinging the mental well-being of young individuals.
